# Evaluation of the effect of perioperative administration of S(+)-ketamine hydrochloride injection for postoperative acute pain in children: study protocol for a prospective, multicenter, randomized, open-label, parallel-group, pragmatic clinical trial

**DOI:** 10.1186/s13063-022-06534-z

**Published:** 2022-07-23

**Authors:** Hong Wang, Chongyang Duan, Jianmin Zhang, Shuangquan Qu, Ying Sun, Lizhi Zhou, Lujia Yang, Chen Lan, Weidong Mi, Pingyan Chen

**Affiliations:** 1 Department of Anesthesiology, The First Medical Center of Chinese PLA General Hospital, Beijing, 100853 People’s Republic of China; 2 Department of Biostatistics, School of Public Health, Southern Medical University, Guangzhou, 510515 People’s Republic of China; 3 Department of Anesthesiology, Beijing Children’s Hospital, Capital Medical University, National Center for Children’s Health, Beijing, 100045 People’s Republic of China; 4 Department of Anesthesiology, Hunan Children’s Hospital, Changsha, 410007 People’s Republic of China; 5 Department of Anesthesiology, Shanghai Children’s Medical Center, School of Medicine, Shanghai Jiao Tong University, Shanghai, 200120 People’s Republic of China

**Keywords:** Postoperative pain, Acute pain, Children, Perioperative period, Anesthesia, Analgesia, S(+)-ketamine, S(+)-ketamine hydrochloride, Esketamine, Ketamine

## Abstract

**Background:**

Inadequate postoperative pain management increases the risk of adverse events after the surgery and aggressive perioperative pain prevention has both short-term and long-term benefits. S(+)-ketamine is an N-methyl-D-aspartic acid (NMDA) receptor antagonist with a strong analgesic effect and can significantly relieve postoperative acute pain and reduce opioid consumption. However, for children, it still needs to be confirmed by large sample clinical studies.

**Methods:**

This is a pragmatic, randomized controlled trial which will evaluate the effect of perioperative administration of S(+)-ketamine hydrochloride injection for postoperative acute pain in children in a pragmatic clinical setting. A total of 3000 children (≤17 years old) undergoing surgery will be included in this protocol. Subjects will be randomized 2:1 to either receive S(+)-ketamine hydrochloride injection or conventional therapy without S(+)-ketamine during the entire perioperative period. The primary endpoints are the area under the receiver operating characteristic (ROC) curve of Face Legs Activity Cry and Consolability (FLACC, 0–7 years old) scale score or Numerical Rating Scale (NRS, 8–17 years old) score within 48 h after surgery, and the consumption of opioids within 48 h after surgery. The secondary endpoints include the time of first use of rescue analgesics after surgery, rescue analgesia rate within 48 h after surgery, anesthesia recovery time, incidence of emergency delirium (for 0-7 years old), changes of anxiety and depression scale scores at 48 h after surgery (for 8-17 years old), incidence of intraoperative adverse events (AEs), and incidence of postoperative AEs and pharmacoeconomic indicators. AEs and serious AEs were recorded to evaluate safety.

**Discussion:**

This trial will be the first pragmatic clinical trial to prospectively assess the effect of perioperative administration of S(+)-ketamine hydrochloride injection for postoperative acute pain in children, which is of great significance to the continuous optimization of clinical anesthesia and analgesia programs for children.

**Trial registration:**

This trial was registered in the U.S. National Institutes of Health ClinicalTrials.gov database (http://clinicaltrials.gov; Registration number: NCT04834427). Registered on 8 April 2021.

## Introduction

According to the revised IASP definition (2020), pain refers to an unpleasant sensory and emotional experience associated with, or resembling that associated with actual or potential tissue damage [1]. In line with Chinese guideline for postoperative pain management, acute pain usually lasts less than 1 month and is often associated with surgical trauma, tissue damage, or some disease. Postoperative pain is acute pain immediately after surgery, including physical pain and visceral pain, usually lasting no more than 3 to 7 days [2]. Studies have shown that postoperative acute pain led to changes in respiratory and circulatory function, endocrine function, and immune function, as well as complications such as atelectasis, pneumonia, hypoxemia, and hypercapnia [3, 4]. If it cannot be controlled at the initial onset, it will develop into chronic pain, and its pain nature can also be transformed into neuropathic pain or mixed pain. This not only affects the treatment effect, functional recovery, and quality of life of patients, but also prolongs the length of hospital stay and increases medical costs [5]. Besides, it has been reported that less than 50% of patients receive adequate postoperative analgesia, and the proportion of postoperative acute pain may be even higher in children undergoing surgery [6]. Therefore, effective and standardized perioperative pain management is very important.

Opioids used to be the only option for postoperative analgesia. However, the application of opioids has been increasingly controversial due to its gastrointestinal side effects, respiratory depression, hyperpathia, tolerance, and addiction [2]. In addition, opioids are less effective in treating certain types of pain, particularly visceral and neuropathic pain, while acute postoperative pain is often a combination that cannot be adequately alleviated by opioids alone [7, 8]. The 2016 American Society of Anesthesiologists (ASA) Guidelines states: “The future of acute pain management may be less opioids of choice” [9]. The 2012 ASA Acute Pain Management Guidelines supports the use of non-opioids throughout the course, including local anesthetics, acetaminophen, non-steroidal anti-inflammatory drugs (NSAIDs), cyclooxygenase-2 (COX-2) selective inhibitors, gabapentin, pregabalin, and ketamine [10]. Although these drugs are not as effective as opioids in analgesia, a growing body of data confirms that their role in perioperative analgesia is more important than known.

S(+)-ketamine is a multi-target drug with the same mechanism of action as racemic ketamine. Ketamine’s analgesic properties in acute pain likely derive from its reversible antagonism of the N-methyl-D-aspartate (NMDA) receptor, although it exerts effects on μ-opioid receptors, muscarinic receptors, monoaminergic receptors, γ-aminobutyric acid receptors, and several others [11, 12]. In the evaluation of efficacy, it is generally believed that S(+)-ketamine has the characteristics of higher potency, stronger receptor affinity, and fewer adverse effects of the nervous system [13–15]. At present, the sub-anesthetic dose of S(+)-ketamine has been widely used in European countries, especially in the field of perioperative acute pain management, which has a good application prospect. Clinical studies have also shown that the sub-anesthetic dose of S(+)-ketamine can effectively relieve postoperative pain, reduce the amount of postoperative analgesics, prolong the analgesic time, relieve opioid drug tolerance, and inhibit hyperpathia, which is a relatively safe and reliable drug [16–18].

According to the clinical use of S(+)-ketamine in the listed countries, the effect of S(+)-ketamine on the prevention of postoperative acute pain in children remains controversial. In addition, the optimal drug regimen of S(+)-ketamine is still unclear, and there are great differences in the dosage, administration method, administration timing, and drug compatibility. Therefore, this study aims to recruit multiple centers to establish the clinical application of S(+)-ketamine by large-sample pragmatic clinical trial, evaluate the efficacy and safety of S(+)-ketamine during the perioperative period, focus on the perioperative acute pain, and explore the effects of S(+)-ketamine on postoperative emergency delirium, postoperative anxiety, and depression mood, as well find the best usage, including dose, timing, compatibility.

## Materials and methods

### Trial design

This prospective, multicenter, randomized, open-label, parallel-group, practical clinical trial will enroll 3000 children from 30 centers in China. This trial is conducted in accordance with Declaration of Helsinki and Good Clinical Practice Guidelines, and approved by ethics committee institutional review board of each participating center. Subjects will be recruited after informed consent is obtained from the subjects and their guardian.

### Objectives

The primary objective of this study is to evaluate the analgesic effect of perioperative administration with S(+)-ketamine hydrochloride injection based on traditional anesthesia strategies on postoperative acute pain in children undergoing surgery. The secondary objectives of this study are (1) to explore the effect of perioperative administration of S(+)-ketamine hydrochloride injection on postoperative emergency delirium, anxiety and depression mood in children undergoing surgery; (2) to evaluate the safety of perioperative administration of S(+)-ketamine hydrochloride injection in children; and (3) to explore the optimal drug regimen of perioperative S(+)-ketamine hydrochloride injection for children, including the influence of administration route, dosage, timing, compatibility, and type of surgery on the end point of the study.

### Inclusion criteria

Inclusion criteria for patients are as follows: (1) age ≤ 17 years old; (2) plan to carry out elective general anesthesia for digestive tract surgery, orthopedic surgery, urological surgery, or ear surgery; (3) ASA physical status I–III; and (4) the patient or his guardian signed the informed consent voluntarily.

### Exclusion criteria

Exclusion criteria for patients are as follows: (1) expected hospital stay of patients < 48h; (2) Patients who are expected to be admitted to the ICU after surgery; (3) patients who are expected to return to the ward with a tracheal tube after surgery; (4) allergic to the active ingredient or excipient of S(+)-ketamine hydrochloride injection; (5) patients with a severe disorder of consciousness or mental system diseases (schizophrenia, mania, bipolar disorder, psychosis, etc.) or cognitive dysfunction; (6) children with congenital heart disease and severe developmental retardation; and (7) patients with any of the following contraindications of S(+)-ketamine: (a) patients with risk of serious rise of blood pressure or intracranial pressure; (b) patients with high intraocular pressure (glaucoma) or penetrating ocular trauma; (c) patients with poorly controlled or untreated hypertension (Resting systolic blood pressure greater than 180 mmHg, or resting diastolic blood pressure greater than 100 mmHg); and (d) patients with untreated or undertreated hyperthyroidism.

### Drop-out criteria

Drop-out criteria for patients are as follows: (1) patients withdrew the informed consent form voluntarily; (2) lost to follow-up, no efficacy and safety data were collected; and (3) the investigator ordered to withdraw, such as the patient returned to the ward with an unexpected tracheal tube.

### Recruitment

SAFE-SK-C is a multi-center research that will recruit from 30 centers and adopt a competitive method for enrollment. The enrollment period of the research is 12 months. The recruitment of subjects will be completed by an anesthesiologist. Since the inclusion criteria and exclusion criteria of the entire study are relatively loose, and the selected hospitals are more capable and authoritative, a large number of subjects will meet the enrollment conditions. Combined with the operation volume of each center, even if the most conservative, it is estimated that each center can reach the minimum number of cases.

### Who will take informed consent?

All the recruited participants will be subsequently evaluated according to the inclusion and exclusion criteria. We guarantee that all participants will be fully informed and understand in detail about the potential benefits and risks of the trial before they sign the informed consent.

### Biological specimens

Not applicable, this trial does not have biological specimens.

### Power and sample size determination

This study hypothesis that S(+)-ketamine hydrochloride could significantly reduce pain scores and/or opioid consumption within 48 h after surgery compared with routine clinical practice. Due to the lack of reference for primary endpoint, the sample size will be evaluated under different effect size assumption. Assuming power is 80%, two-sided significant level 0.025, and the ratio of S(+)-ketamine hydrochloride group to conventional group is 2:1, the sample size under different effect sizes is shown in Table 1. As shown, about 687 patients will be powerful enough to detect an effect size of 0.25. The effect size d is defined as *d* = (μ1–μ2)/σ, and Cohen (1988) gave the interpretation of d=0.25 as 25% of σ and defines *d* = 0.2, 0.5, and 0.8 as small, medium, and large effect size. In our study, the used effect size of 0.25 is a relatively small effect size. By considering 4 types of surgery, we decide to include 3000 patients. Patients will stratify by age and surgical approach, and enrolled in a competitive enrollment way until the total number of research centers reached the expected sample size.

**Table 1:** The sample sizes under different effect sizes

Control group (*N*)	S(+)-ketamine hydrochloride group (*N*)	Total subjects (*N*)	Effect sizes
58	116	174	0.500
130	260	390	0.333
229	458	687	0.250
357	714	1071	0.200
514	1028	1542	0.167
699	1398	2097	0.143
913	1826	2739	0.125
1155	2310	3465	0.111
1426	2852	4278	0.100

Sample size under different effect sizes assuming power is 80%, two-sided significant level of 0.025, and the ratio of S(+)-ketamine hydrochloride group to conventional group is 2:1

### Randomization and masking

To ensure the comparability of groups, a stratified block randomization method with stratified factors for age and different surgical methods was adopted. An independent statistician generated the stratified permuted block randomization sequence by using SAS software (version 9.4, SAS Institute, USA). Centralized randomization was conducted by using the Interactive Voice Response service provided by Shang Hai Ashermed healthcare communications co., Ltd. Subjects meeting the criteria will be randomly assigned to the trial group or routine clinical practice group (control group) in a 2:1 ratio. The trial group will be administrated with S(+)-ketamine hydrochloride injection and the control group will receive conventional therapy without S(+)-ketamine hydrochloride injection. The most common usage in conventional therapy is propofol combined with an opioid.

The patients will be randomly allocated via an interactive response technology (IRT) system which is independent of the trial team. For each patient, nobody has any access to known the intervention that will be received during the screening stage. Block randomization will be done at the individual level with a randomization list generated by the IRT provider and will be stratified by age and different surgical types. The trial manager will inform the participant after notification of intervention allocation.

Since this is an open-label pragmatic trial, the investigators, the subjects and the sponsors who give intervention cannot be masked from intervention allocation. Notably, the investigator and pain score assessor is not the same one in this trial. The pain score assessor will not be informed of the grouping situation.

### Implementation

The investigators enroll all participants. After signing the informed consent form, each participant will be allocated a sequential number that randomizes them to one of the two groups. All investigators should have the professional expertise, qualifications, and abilities to undertake this clinical research, and will be trained in GCP regulations. Each center should have experience in drug research, and the facilities and conditions of the department where the project is located should meet the needs of safe and effective clinical research. Besides, the investigators will develop the SOP to keep the implementation standards consistent across centers. Meanwhile, in the investigator’s plan to establish Quality Control Inquiry Committee and Data Monitoring Committee, each committee is responsible for the corresponding affairs and ensures the quality. Besides, we will cooperate with Contract Research Organization, and CRA will be responsible for monitoring and ensuring that the project implementation conforms to GCP.

### Intervention

In the control group, subjects will receive conventional therapy without S(+)-ketamine. There are no restrictions on drugs, doses, and incompatibility; the researchers can choose appropriate medication regimens based on clinical practice, but other NMDA receptor antagonists will not be allowed to use, such as dextromethorphan and amantadine.

In the trial group, S(+)-ketamine hydrochloride will be administrated for general anesthesia for anesthesia induction, maintenance, or postoperative analgesia. In principle, there are no specific restrictions on the dosage, way of administration, timing, and compatibility of S(+)-ketamine hydrochloride injection, but the recommended dosage is given. The recommended dose range for intravenous injection (1) for intravenous injection is as follows: (a) bolus intravenous injection before skin incision, the dose is 0.1–0.25 mg/kg; (b) bolus intravenous injection (dose 0.1–0.25 mg/kg) before skin incision and continuous intravenous infusion (dose of 0.1–0.25 mg/kg/h) during operation; and (c) continuous intravenous infusion after surgery with a dose of 0.02–0.1 mg/kg/h for 24–48 h and (2) for intramuscular injection as the dose is 2–4 mg/kg.

### Strategies to improve adherence to interventions

At the beginning of the trial, participants will be informed of the experiment procedure again. They can report their personal feelings at any time during the study, and we will give corresponding feedback as appropriate to increase the interactivity and then improve adherence to interventions.

### Clinical assessment

The primary endpoints are (1) the area under the receiver operating characteristic (ROC) curve of the FLACC scale score or Numerical Rating Scale score within 48 h after surgery (age ≤ 7 years old, the FLACC scores at 10 min, 20 min, 30 min, 2 h, 4 h, 24 h, and 48 h after surgery will be calculated; 8 ≤ age ≤ 17 years old, NRS scores at 2 h, 4 h, 24 h, and 48 h after surgery will be calculated); and (2) the consumption of opioids within 48 h after surgery, including the sum of all opioids consumed within 48 h after surgery.

The secondary endpoints are (1) FLACC pain scores at 10 min, 20 min, 30 min, 2 h, 4 h, 24 h, and 48 h after surgery (age ≤ 7 years old); (2) NRS pain scores at 2 h, 4 h, 24 h, and 48 h after surgery (age ≤ 17 years old); (3) time to first rescue analgesics after surgery; (4) the incidence of rescue analgesia within 48 h after surgery; (5) anesthesia recovery time; (6) the incidence of emergence delirium (age ≤ 7 years old, pediatric anesthesia emergence delirium (PAED) will be used to evaluate the delirium from anesthesia in children, which will be evaluated at 10 min, 20 min and 30 min after surgery); (7) the incidence of unexpected intraoperative events (including intraoperative cough, laryngeal spasm, body movement, tachycardia, decreased oxygen saturation, respiratory depression, bradycardia, hypertension, and hypotension); (8) the incidence of adverse events after surgery (including nausea, vomiting, increased secretions, dizziness, over sedation, infection, anesthetic awareness, nightmares, restlessness, pruritus, disorientation, delirium, and respiratory depression); (9) changes of anxiety and depression scale scores at 48 h after surgery relative to baseline (8 ≤ age ≤ 13, depression will be assessed using the Children’s Depression Inventory (CDI); 14 ≤ age ≤ 17, depression will be assessed using the CDI and Hospital Anxiety and Depression Scale (HAD)); and (10) pharmacoeconomic indicators (incremental cost-effectiveness ratio (ICER) based on cost-effectiveness analysis). Intraoperative pain is usually monitored by fluctuations in the patient's blood pressure and heart rate. Besides, breakthrough analgesics during the intra-operative period and how will this impact the outcome of the study will be specially reported in the Electronic Data Capture System (EDC).

### Safety assessments, reporting, and harms

Safety of treatment regimens will be assessed by the changes of vital signs, physical examinations, laboratory data, the incidence of adverse events (AEs), and serious adverse events (SAEs). AEs are defined as any unexpected medical event experienced by subjects after drug treatment. It can be manifested as symptoms, signs, diseases, or abnormal laboratory tests, but it is not necessarily inferred that there is a clear causal relationship with experimental drugs. SAEs are defined as any of the following: death, life-threatening events, requirement of hospitalization and prolonged hospitalization, deformities and birth defects, significant/permanent disabilities or organ damages, and important medical events or intervention measures.

Both AEs and SAEs that occurred during drug therapy will be recorded on the case report form (CRF) accordingly. SAEs that are considered related to the study are monitored and reported without delay. Study-specific reportable adverse events, including death, or resulting in a life-threatening situation or significant disability, or requiring inpatient hospitalization or prolongation of existing hospitalization are also being monitored during the trial.

Common AEs include (1) increase of blood pressure and heart rate, and generally do not need to be treated. If necessary, esmolol can be used to relieve (2) increase of secretions, use a negative pressure catheter to suck out the secretions, or use atropine to prevent; (3) blurred vision and diplopia, they are transient and generally do not need to be dealt with; (4) agitation, delirium, separation anxiety, and other psychiatric adverse reactions, they are transient reactions, which can recover spontaneously in the short term without special treatment. Psychological counseling can be given, and benzodiazepines can be given when necessary; (5) nausea and vomiting, generally mild and tolerable, may be alleviated by the use of antiemetics such as Setron, if necessary.

### Ancillary and post-trial care

At the end of the study, we will provide a certain amount of subsidy to each subject. If the adverse events occur during the trial that are related to the intervention after the expert committee confirms, the responsible department will provide corresponding economic compensation, psychological comfort, and treatment modalities. Before the trial, the participants will be informed that participation in this study is voluntary, they can withdraw from the study at any time for any reasons, and their benefits will not be affected.

### Participant timeline

**Fig. 1:**
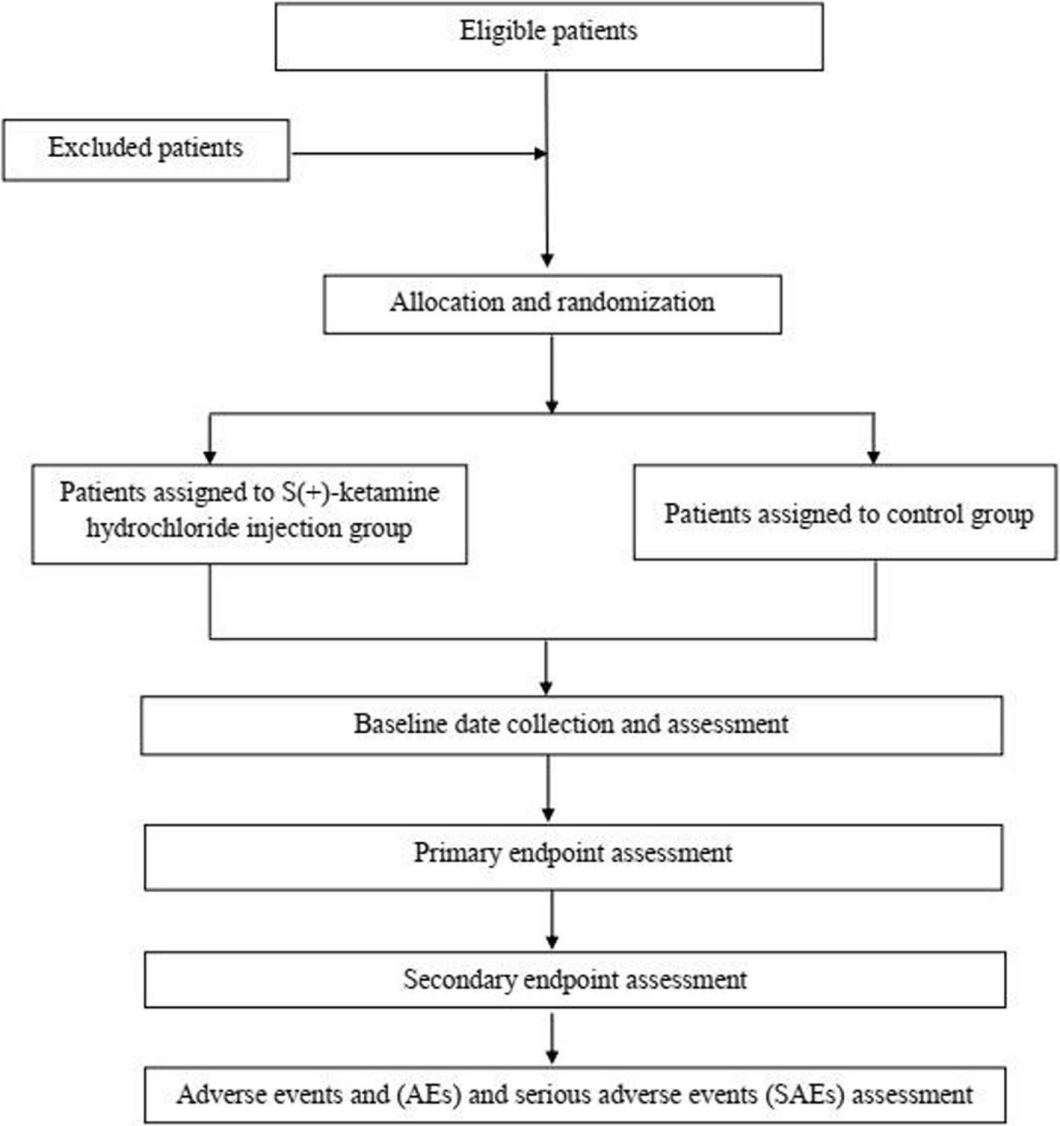
Participant flow

After the subjects signed the informed consent, all subjects meeting the criteria will include in this study, stratified according to age and type of surgery. Clinical and demographic variables will be collected and reported including age, gender, blood tests, blood coagulation, urine routine, liver and kidney function, ECG, disease-related information (such as allergy history, present medical history, previous medical history, combined disease and concomitant medication), FLACC score, NRS score, AEs, and SAEs. After randomization, patients will be assessed on the screening period, preoperative, intraoperative, and at postoperative 10 min, 20 min, 30 min, 2 h, 4 h, 24 h, and 48 h. The participant flow is shown in Fig. 1. A time schedule for enrolment, interventions, and assessments is presented in Tables 2 and 3.

**Table 2:** Study schedule of assessments (age ≤ 7 years old)

Item	Screening	Follow-up								
	V0	V1						V2		V3
	Preoperative	Preoperative	Intraoperative	Postoperative				Postoperative		Postoperative
	−7–0 days			10 min	20 min	30 min	2 h	4 h	24 h±3 h	48 h±3 h
Signing informed consent form	√									
Randomization		√								
Basic information	√									
Inclusion/exclusion criteria	√									
Basic medical history	√									
Surgical information	√		√							
vital signs	√	√	√							
Laboratory examination	√								*	*
ECG examination	√								*	*
FLAAC score	√			√	√	√	√	√	√	√
Analgesic dosage			√						√	√
PEAD evaluation				√	√	√				
Concomitant medication	√		√						√	√
AEs			√	√	√	√	√	√	√	√
SAEs			√	√	√	√	√	√	√	√

*ECG* electrocardiogram, *FLAAC* Face Legs Activity Cry and Consolability, *PEAD* Pediatric Anesthesia Emergence Delirium, *AEs* adverse events, *SAEs* serious adverse events

√: required; ^*^: optional

Laboratory examination: blood routine, blood biochemical index, urine routine, and coagulation function

**Table 3:** Study schedule of assessments (8 ≤ age ≤ 17 years old)

Item	Screening	Follow-up					
	V0	V1				V2	V3
	Preoperative −7–0 days	Preoperative	Intraoperative	Postoperative		Postoperative	Postoperative
				2 h	4 h	24 h±3 h	48 h±3 h
Signing informed consent form	√						
Randomization		√					
Basic information	√						
Inclusion/exclusion criteria	√						
Basic medical history	√						
Surgical information	√		√				
Vital signs	√	√	√				
Laboratory examination	√					*	*
ECG examination	√					*	*
NRS score	√			√	√	√	√
Analgesic dosage			√			√	√
Depression/anxiety evaluation^#^	√						√
Concomitant medication	√		√			√	√
AEs		√	√	√	√	√	√
SAEs		√	√	√	√	√	√

*ECG* electrocardiogram, *NRS* Numeric Rating Scale, *AEs* adverse events, *SAEs* serious adverse events

√: required; ^*^: optional

Laboratory examination: blood routine, blood biochemical index, urine routine, and coagulation function

^#^: 8 ≤ age ≤ 13 years old, depression/anxiety will be assessed using the Children’s Depression Inventory (CDI); 14 ≤ age ≤ 17 years old, depression/anxiety will be assessed using the CDI and Hospital Anxiety and Depression Scale (HAD))

### Data collection

Data will be collected using the EDC system and mobile APP terminal. Trial processes will be embedded within the electronic case report form (eCRF) to improve trial efficiency. Data collection will divide into two stages. In the first stage, each cooperative unit will collect 5% of the total number of cases in charge to familiarize themselves with the platform, discover problems, and cooperate with the research undertaking unit to prepare the materials required for verification. According to the feedback and data verification of the cooperative units, adjust CRF, challenge the medical records with problems, and supervise the cooperative units to correct them. In the second stage, all the remaining cases will be recorded.

### Data verification, cleaning, and locking

The datasets used and/or analyzed during the current study will be made available from the corresponding author upon reasonable request. The database will be locked immediately after all data are entered and all discrepant or missing data are resolved, or, alternatively, if all efforts have been employed and we consider that the remaining issues cannot be fixed. In this step, the data will be reviewed before database locking. The study database will then be locked and exported for statistical analysis. At this stage, permission for access to the database will be removed for all investigators, and the database will be archived.

### Data management

Source documents will save in a secured and logged Sharepoint and keep by each center in case of medical reports. Documents specific to the trial will be archived by the investigator and the sponsor for 15 years following the end of the study in accordance with national guidelines. Results from the protocol-specific procedures will be collected in paper format and then entered into the eCRF. The assessors enter all other data directly into the eCRF during the visits. Secured login and password will be attributed by a data manager. Data quality in the eCRF will be promoted using range checks for data values. Each missing data item must be coded. Data will later be transferred to a statistical program for analyses. The data will be anonymized 5 years after the termination of the study.

### Non-adherence and dropout data

Participation in the trial is voluntary, and patients will have the right to withdraw consent at any time, for any reason, without any consequence for further medical treatment. All data in this study will be analyzed according to the principle of intention-to-treat (ITT) to reduce the deviation. The reasons and circumstances for study discontinuation will be documented in the CRF. The patients’ participation in this study can also be ended by the investigator if the patient is uncooperative and/or does not attend the study visits. In this case, patient data collected up until the point of removal will be included in the analysis; if too many data are missing (e.g., missing baseline, study visits, and other measurements included in the primary outcome), the patient will be replaced by a new patient. Patients who convert to open surgery will be treated according to the standard of care, based on the findings during surgery. These patients will be excluded from the study, and patient data until this moment will be included in the analysis.

### Confidentiality

All data about potential and enrolled participants will be collected in a secure and logged database, in a secure and logged Sharepoint, or behind a double lock for data in paper format. Only anonymized data will be shared.

### Plans to give access to the full protocol, participant-level data, and statistical code

The data sharing process will comply with the principles of good practice, and the data sharing will be conducted in accordance with the regulatory requirements.

### Auditing

Investigators will cooperate with Contract Research Organization, and CRA will be responsible for monitoring and ensuring that the project implementation conforms to GCP.

### Statistical analysis

All statistical analyses will be performed using SAS 9.4 (SAS Institute Inc., USA). All data in this study will be analyzed according to the principle of ITT to reduce the deviation. The categorical data is expressed as numbers (%) and frequency and will be compared with Pearson chi-square or Fisher’s exact test. Continuous data will be expressed as mean ± standard deviations (SD) or median, minimum, maximum, interquartile range (IQR) and will be compared by Student’s *t* test and Wilcoxon rank-sum test.

For the primary endpoints, covariance analysis (ANCOVA) will be used to perform the between-group comparisons and the age and surgery types that are used as stratification factors in the randomization will be included as covariables. The FLACC scale score and NRS score will be analyzed separately, and then the standardized mean differences (SMD) will be combined by using the fixed effect meta-analysis model. To deal with the multiplicity problem caused by the two primary endpoints, the significance level will set be two-sided 0.025 by using the Bonferroni method. In subgroup analysis, according to age, type of surgery, intervention methods (such as drug administration method, dosage, type of other analgesics, presence or absence of nerve block, etc.) and possible combinations (combination plan of study drug and other drugs), subgroup analysis of the primary endpoints will be performed. For secondary endpoints, univariate analysis and multivariate analysis (Generalized Linear Mixed Model (GLMM)) will be used to perform the between-group comparisons. For safety analysis, the Pearson *χ*^2^ test or Fisher’s exact test will be used for comparison between groups. Effect sizes will be reported with 95% confidence intervals. All tests will be two-sided and the significance level is set as 0.025.

### Interim analyses

Not applicable. Interim analyses will not be performed.

### Dissemination plans

Information about the trial is published at the U.S. National Institutes of Health clinicaltrials.gov database (http://clinicaltrials.gov) before enrolment of the first patient. The protocol and study results will be published in international peer-reviewed journals. Both positive, negative, and inconclusive results will be published. After publication, the results from the trial will be disseminated to the trial participants via email and to the public via written and Internet media.

### Composition of trial organization, its role, and reporting structure

Sponsor: Chinese PLA General Hospital.

Collaborators: Southern Medical University; Beijing Children’s Hospital, Capital Medical University; Shanghai Children’s Medical Center, School of Medicine, Shanghai Jiao Tong University; Hunan Children’s Hospital; Children’s Hospital of Shanghai; The First Affiliated Hospital of Shantou University Medical College; Jiangxi Provincial University Hospital; Binzhou Medical University Hospital; Affiliated Nanhua Hospital, University of South China; The First Affiliated Hospital of Shaoyang University; Kunming Children’s Hospital Yueyang First People’s Hospital; The First People’s Hospital of Chenzhou; Guiyang Maternal and child Health Care Hospital; Children’s Hospital, Capital Institute of Pediatrics; Guangdong Province Maternal and child Health Hospital; Henan Children’s Hospital; Dalian Women and Children’s Medical Center; Guangxi Maternal and child Health Hospital; Henan Provincial People’s Hospital; Meizhou People’s Hospital; Sun Yat-sen University Cancer Center; The First Affiliated Hospital of Xinjiang Medical University; Taizhou Central Hospital; The Children’s Hospital, Zhejiang University School of Medicine; The First Hospital of Changsha; Sichuan Provincial Maternity and Child Health Hospital; Children’s Hospital affiliated to Fudan University.

The trial is managed by a team consisting of the chief investigators (Weidong Mi, Pingyan Chen, Hong Wang and Chongyang Duan), the trial coordinators (Jianmin Zhang and Shuangquan Qu), statisticians, the informatics technician responsible for the web-based electronic data capture system, and independent monitors. A steering committee (The First Medical Center of Chinese PLA General Hospital) contributed to the design and revision of the study and will be responsible for the interpretation of data and compilation of the resulting manuscript. Patient data and safety will be closely monitored by the Southern Medical University. All AEs entered into the eCRF within prespecified time frames, including severe AEs and suspected unexpected severe adverse reactions, will be monitored by an AE manager (Jianmin Zhang and Shuangquan Qu), who will provide reports for review. The coordinator will be responsible for administration and the assistance during trial management and data collection.

## Discussion

More than 80% of patients who undergo surgical procedures experience acute postoperative pain and approximately 75% of those with postoperative pain report the severity as moderate, severe, or extreme [6]. Inadequate postoperative pain management increases the risk of adverse events after the surgery and is the main risk factor related to chronic post-surgical pain [4]. Therefore, positive perioperative pain prevention and perioperative analgesia have attracted more and more attention and attention from doctors and patients. Many preoperative, intraoperative, and postoperative interventions and management strategies are available and continue to evolve for reducing and managing postoperative pain [2]. The American Pain Society (APS) commissioned a guideline on the management of postoperative pain to promote evidence-based, effective, and safer postoperative pain management in children and adults, addressing areas that include preoperative education, perioperative pain management planning, use of different pharmacological and nonpharmacological modalities, organizational policies and procedures, and transition to outpatient care. Ketamine has been recommended as an important member of multimodal analgesia [9, 10].

S(+)-ketamine is the dextrorotatory isomer of ketamine, which is safer and suitable in induction and maintenance of general anesthesia, as a supplement to local anesthesia, anesthesia in children, and anesthesia and analgesia in first aid. In recent years, the perioperative analgesia of S(+)-ketamine is also an important direction, some of the studies have shown its effects in analgesia and hyperpathia. However, there are great differences in the optimal dosage, administration method, administration timing, and drug compatibility, and there are few studies in children [19–21]. BECKE et al. showed that intraoperative 0.2 mg/kg^−1^ S(+)-ketamine had additional analgesic and sedative effects on children with major urological surgery in children [22]. Brinck et al. compared two different doses (0.12 mg/kg/h or 0.6 mg/kg/h) of S(+)-ketamine on postoperative analgesia in opioid-naïve patients and found that although both doses reduced the pain score, they did not reduce the consumption of opioids [23]. Ciment et al. evaluated the effects of minimal-dose (0.125 mg/kg/h) and low-dose (0.015 mg/kg/h) S(+)-ketamine on opioid consumption, hyperalgesia, and postoperative delirium. The data revealed minimal-dose S(+)-ketamine was comparable to the conventional low-dose regimen in reducing postoperative opioid consumption and hyperalgesia. However, Postoperative delirium was less frequent with the minimal-dose regimen [24]. In addition, we focused on the effects of different administration modes on S(+)-ketamine analgesia. In Martindale et al.’s study, they demonstrated that the addition of caudal S(+)-ketamine to bupivacaine prolongs the duration of postoperative analgesia. However, the same dose of i.v. S(+)-ketamine combined with a plain bupivacaine caudal provides no better analgesia than caudal bupivacaine alone [25]. Nemeth et al proved the intranasal administration for analgesia and/or sedation was a reasonable delivery route in pediatric acute care, when intravenous access was impossible or inappropriate [26]. Besides, Koinig et al. also compared the analgesic effects of caudal and intramuscular S(+)-ketamine in children [27]. In terms of drug combinations, Nemeth et al showed a fentanyl-, s-ketamine-, and midazolam-based therapeutic regimen was effective and safe for acute pain [26]. WEBER et al depicted that the addition of S(+)-ketamine to caudal bupivacaine provided significant prolongation of analgesia without producing negative side effects [28].

This trial will be the first pragmatic clinical trial with a sufficient sample size to prospectively assess the effect of perioperative administration of S(+)-ketamine hydrochloride injection for postoperative acute pain in children. In addition, this trial follows the principles of ITT, through stratified randomization, sensitivity analysis, and subgroup analysis to reduce the risk of bias and provide high-quality evidence. Finally, the transparency of the applied methods and definitions of outcome measures will be ensured through public access to the current protocol paper and a priori registration at http://clinicaltrials.gov. This research is of great significance to the continuous optimization of clinical anesthesia and analgesia programs for the children population.

### Trial status

 Registration date: April 8, 2021.

Estimated Study Start Date: May 1, 2021.

Estimated Primary Completion Date: December 1, 2022

Estimated Study Completion Date: December 30, 2022.

## Data Availability

The datasets supporting the conclusions of this article are included within the article (and its additional files).

## Abbreviations

NMDA: N-methyl-D-aspartic acid
AUC: Area under the broken line
FLACC: Face Legs Activity Cry and Consolability
NRS: Numerical Rating Scale
AE: Adverse events
SAE: Serious adverse events
NSAIDs: Non-steroidal anti-inflammatory drugs
COX-2: Cyclooxygenase-2
